# In Situ Growth of Nano-MoS_2_ on Graphite Substrates as Catalysts for Hydrogen Evolution Reaction

**DOI:** 10.3390/ma16134627

**Published:** 2023-06-27

**Authors:** Yifan Zhao, Mingyang Zhang, Huimin Zhao, Zhiqiang Zeng, Chaoqun Xia, Tai Yang

**Affiliations:** 1 School of Materials Science and Engineering, Hebei University of Technology, Tianjin 300130, China; zhaoyifan1020@163.com (Y.Z.); hebutyf@163.com (M.Z.); 16622813573@163.com (H.Z.); zzq970427@126.com (Z.Z.);; 2 Tianjin Key Laboratory of Laminating Fabrication and Interface Control Technology for Advanced Materials, Hebei University of Technology, Tianjin 300130, China

**Keywords:** hydrogen evolution reaction, catalyst, graphite, MoS_2_, in situ deposition

## Abstract

In order to synthesize a high-efficiency catalytic electrode for hydrogen evolution reactions, nano-MoS_2_ was deposited in situ on the surface of graphite substrates via a one-step hydrothermal method. The effects of the reactant concentration on the microstructure and the electrocatalytic characteristics of the nano-MoS_2_ catalyst layers were investigated in detail. The study results showed that nano-MoS_2_ sheets with a thickness of about 10 nm were successfully deposited on the surface of the graphite substrates. The reactant concentration had an important effect on uniform distribution of the catalyst layers. A higher or lower reactant concentration was disadvantageous for the electrochemical performance of the nano-MoS_2_ catalyst layers. The prepared electrode had the best electrocatalytic activity when the thiourea concentration was 0.10 mol·L^−1^. The minimum hydrogen evolution reaction overpotential was 196 mV (*j* = 10 mV·cm^−2^) and the corresponding Tafel slope was calculated to be 54.1 mV·dec^−1^. Moreover, the prepared electrode had an excellent cycling stability, and the microstructure and the electrocatalytic properties of the electrode had almost no change after 2000 cycles. The results of the present study are helpful for developing low-cost and efficient electrode material for hydrogen evolution reactions.

## 1. Introduction

Renewable energy is seen as a powerful way to reduce global carbon emissions, and it will become an integral part of energy in the future [1]. Sustainable, cheap, safe, and clean energy sources are a research hotspot in the pursuit of a green economy and due to increasing demands for energy [2,3]. Solar, wind, geothermal, bioenergy, and hydrogen energies are among the types of renewable energy [4]. Of these, hydrogen energy is clean, green, and sustainable and has a high energy density. It is also easy to produce. The evolution of hydrogen from natural gas is the common approach in current hydrogen-evolution methods, but reserves are insufficient [5]. When coal is used to produce hydrogen, CO and CO_2_ are generated during the reaction process, polluting the environment [6]. In the biological hydrogen evolution process, many impurities are generated, and purification is difficult [7]. Photocatalytic hydrogen evolution is a promising method, but there are still some unresolved issues. The instability of photocatalysts and the low efficiency of energy conversion limit its application [8,9,10]. 

The reaction conditions for electrocatalytic hydrogen evolution are mild, and no impurities are produced during the preparation process. Electrocatalytic hydrogen evolution has received much attention because of its virtuous cycle and its ease of operation [11,12]. In this case, the process of hydrogen preparation using electrolysis of water is considered to be a feasible approach [13]. To improve the efficiency of electrocatalysis for water decomposition, there is an immediate requirement to develop effective and reliable catalysts to expedite hydrogen evolution reaction (HER) dynamics [14,15]. Platinum is the most important HER catalyst owing to its suitable free energy of hydrogen adsorption [16]. However, as a noble metal catalyst, platinum is expensive and scarce, which limit its wide application [17,18]. Therefore, the development of low-cost and high-activity electrocatalysts is urgently needed [19,20].

Transition metal sulfides have become important materials due to their high-potential HER performance and low cost [21,22]. The sulfur element in transition metal sulfides can modulate the electronic properties of metals and improve their catalytic activity. Metal sulfides such as CoS_2_, NiS_2_, and MoS_2_ have been shown to have good HER activity. Among them, CoS_2_ has been widely studied because of its rich composition, semimetallic conductivity, large surface area, and significant catalytic activity. Importantly, CoS_2_ has low chemisorption energy for hydrogen, which facilitates hydrogen generation [23]. Zhang et al. [24] used a hydrothermal method to deposit CoS_2_ on titanium foil and showed that an extended treatment time of the hydrothermal reaction induced pyramidal CoS_2_ morphology formation. The catalyst of the hydrothermal reaction at 15 h showed a very small onset potential of about 81 mV. Pyrite-based NiS_2_ was reported to have unlimited potential to be widely used in hydrogen precipitation reactions due to its low cost, good stability, and ease of preparation [25,26]. Ma et al. [27] synthesized two-dimensional nickel disulfide grown on nickel foam with an average thickness of 10 nm and an overpotential of 161 mV at a current density of 100 mA·cm^−2^. WS_2_ has significant electrochemical activity due to its rapid diffusion of ions, easily accessible edge site structure, and large surface area [28]. Duan et al. [29] delaminated bulk WS_2_ to obtain 2D WS_2_ nanolayers and then doped the WS_2_ nanolayers into graphene films. The catalyst exhibited extraordinary HER performance, with a high current density and long-lasting stability, because the 2D WS_2_ surface area and the exposed edges were significantly increased, which was very favorable for HER.

MoS_2_ is a typical two-dimensional material [30,31]. Like graphite, it features a lamellar structure, with layers bonded internally by strong covalent bonds and layers connected through weak van der Waals forces [32]. It can be easily peeled off between layers an thus, has excellent properties for catalysis and lubrication. For these reasons, this new material has attracted much attention in recent years [33,34]. Therefore, MoS_2_ is often used as a catalyst for photocatalytic and electrocatalytic hydrogen evolution. Lin et al. [35] prepared a 10 nm thick MoS_2_ monolayer via a stripping method. The nanoscale MoS_2_ monolayer had a larger contact area, which improved the activity of the catalyst in the photocatalytic reaction. Fu et al. [36] prepared PCN/MoS_2_ via an ultrasonic dispersion method. The band structure of PCN and MoS_2_ was staggered, which improved the separation rate of the photocarrier an thus, improved photocatalytic efficiency. However, MoS_2_, in its pure form, lacks catalytic activity. The inherent low electrical conductivity inhibits effective electron transfer and the associated electrochemical kinetics, affecting the speed of HER [37]. To improve the electrocatalytic properties of MoS_2_, researchers have used various methods [38]. A novel nanostructure with an increased surface area is an effective way to increase the number of exposed edges. Nguyen et al. [39] developed a hybrid material with a mesoporous nanosheet heterostructure, and a unique hollow core–shell structure with strong electronic interactions between the core part and the core shell was obtained. The electronic structure of MoS_2_ can be effectively tuned by introducing heteroatoms in the MoS_2_ base plane. Tang et al. [40] prepared Fe-hybridized MoS_2_ nanosheets and found that doping with Fe not only affected the synthesis processes of MoS_2_ nanosheets but also optimized the edge sites and the electronic properties of MoS_2_.

To date, most of the prepared HER catalytic agents have been sold in powder form. When performing electrocatalytic reactions, powdered electrocatalysts require Nafion as a binder to drip-coat catalysts on a conductive carrier. Powdered samples can suffer from flaking and loss during electrocatalysis, leading to errors in experimental testing. In addition, adhesive addition can reduce the conductivity of an electrode. Directly depositing nanometer catalysts on a conducting material is an effective way to solve the problem. When a graphite substrate is used as the reaction substrate, there is direct contact between catalyst and electrolyte, and their contact area is increased. Additionally, there is no need to use Nafion as binder, and the conductivity of electrodes is not affected. This method optimizes the electrode preparation process and reduces the experimental cost.

Therefore, in the current study, we worked on preparing an effective and economical HER electrocatalyst for MoS_2_ deposition in situ on a graphite substrate. The graphite substrate was used as a reaction substrate material to avoid catalyst dropout during hydrogen evolution. Moreover, this method reduced MoS_2_ nanosheet buildup and exposed more edges to accelerate electron transfer, hence greatly improving the overall conductivity of the HER electrode.

## 2. Experimental Details

### 2.1. Materials

Sodium molybdate (Na_2_MoO_4_·2H_2_O) and thiourea (CH_4_N_2_S) were purchased from Tianjin Kemeiou Chemical Reagent Co., Ltd. (Tianjin, China). Nitric acid was purchased from Tianjin Fuchen Chemical Reagent Co., Ltd. (Tianjin, China). Graphite plates with a thickness of 2 mm were obtained from Zibo Baofeng Graphite Co., Ltd. (Zibo, China). The materials used in the experiments did not require additional treatment. To reduce the risk of sample contamination, deionized water was used throughout the experimental procedure.

### 2.2. Sample Preparation Method

Graphite plates were cut into a size of 10 mm × 10 mm, leaving a 3 mm wide section on one side for electrode clamping. We sanded the cut graphite plates with sandpaper until the surfaces were shiny and free of scratches. Then graphite plates were ultrasonicated in concentrated nitric acid for 2 h. After washing and drying, the acid-corroded graphite plates could be used as a substrate for depositing nano-MoS_2_ catalyst.

The working electrodes that nano-MoS_2_ in situ deposited on graphite substrates (MoS_2_@Gr) were prepared via a one-step hydrothermal method. The mixed solution for the hydrothermal process was prepared by ultrasonically dispersing Na_2_MoO_4_·2H_2_O and CH_4_N_2_S in 60 mL of deionized water until completely dissolved. One graphite substrate was vertically placed in a 100 mL reaction vessel and subjected to hydrothermal reaction at 200 °C for 24 h. When finished with the reactions, products were cleaned with deionized water and ethanol for 5 min and then dried at 60 °C in a vacuum drying oven. Five different reactant concentrations were used in this study. The concentration of thiourea was 0.05, 0.07, 0.10, 0.12, or 0.15 mol·L^−1^; the concentration of Na_2_MoO_4_ was one-third of that of thiourea. For convenience, the products with different thiourea concentrations were named MoS_2_@Gr-0.05, MoS_2_@Gr-0.07, MoS_2_@Gr-0.10, MoS_2_@Gr-0.12, and MoS_2_@Gr-0.15, respectively. In addition, pure MoS_2_ powders were prepared under the same synthesis conditions with a thiourea concentration of 0.10 mol·L^−1^, and this product was labeled MoS_2_.

### 2.3. Structural Characterizations

The microscopic appearance of the MoS_2_@Gr samples was determined with a scanning electron microscope (SEM, JEM-7100F, JEOL Ltd., Akishima, Japan). Structural properties of the electrodes were characterized via X-ray diffraction (XRD) using a Bruker D8 Discover-type powder diffractometer (Bruker, Billerica, MA, USA) with Cu-K_α_ radiation. The elemental composition and valence bonding state of electrode surface were determined with an ESCALAB 250Xi X-ray photoelectron spectrometer (XPS) (Thermo Fisher Scientific, Waltham, MA, USA). In addition, a confocal Raman spectrum analyzer with an excitation wavelength of 532 nm for Raman analysis was applied. Contact angle measurement of surface wettability was performed using a contact angle meter with a camera (Powereach, JC2000, Shanghai, China). Atomic force microscopy (AFM, NYSE: A; Agilent, Santa Clara, CA, USA) was used to measure the roughness and surface area of graphite.

### 2.4. Electrochemical Tests

An electrochemical workstation (CHI660E) was selected for the electrochemical tests. A saturated calomel electrode (SCE) was used as a standard reference electrode, a platinum mesh served as a counter electrode, the prepared MoS_2_@Gr samples were directly treated as the working electrode, and 0.5 M H_2_SO_4_ solution was substituted as the electrolyte. Electrochemical tests of pure MoS_2_ powders were performed with the aid of a glassy carbon electrode, and the test electrode was obtained by referring to the preparation method of Wang et al. [41]. All the tests were carried out at room temperature. During electrochemical testing, *E*_SCE_ was calibrated with a reversible hydrogen electrode (RHE) and converted to *E*_RHE_, where *E*_RHE_ = *E*_SCE_ + 0.059 pH + 0.241 V. Linear sweep voltammetry (LSV) was tested over a range of −0.6~0 V (vs. RHE), and the sampling rate was 2 mV·s^−1^. The electrochemical impedance spectra (EIS) were measured in a frequency range of 100 kHz to 0.01 Hz with an AC voltage of 5 mV. To measure the double-layer capacitance (*C*_dl_) of each electrode, cyclic voltammograms (CV) with different sweep rates (10, 20, 40, 60, 80, 100, or 120 mV·s^−1^) were collected in a voltage range of 0.15~0.35 V (vs. RHE). Chronoamperometry measurements were also recorded at an overpotential of 200 mV, and changes in polarization curves were compared after 2000 cycles of CV tests to investigate the stability of the catalyst.

## 3. Results and Discussion

### 3.1. Morphology and Structure of MoS_2_@Gr Samples

**Figure 1:**
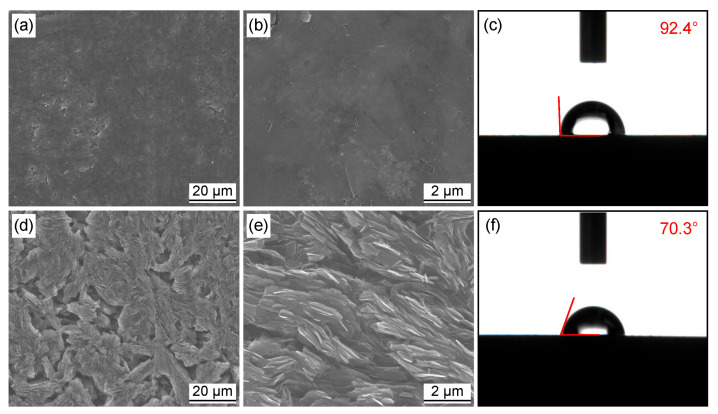
(**a**–**c**) SEM images and contact angle of polished graphite substrate; (**d**–**f**) SEM images and contact angle of acid-corroded graphite substrate.

Graphite is very suitable for electrodes due to its high conductivity and low cost. However, its disadvantages are its low surface tension, a wide area without defects, fewer oxygen-containing functional groups on the surface, and poor hydrophilicity. There is no force between the catalyst and graphite substrate, which is not conducive to the adsorption of transition metal ions. Therefore, graphite substrates were first modified using acid corrosion to increase the surface area and improve the hydrophilicity of the substrate surfaces. Figure 1 shows the SEM images and contact angle of the polished and acid-corroded graphite substrate. It can be seen from Figure 1a that the polished graphite substrate surface was relatively smooth, and some small mottling existed on the substrate surface. This was because the graphite layers were not firmly bonded together during the pressing and sintering process. The water contact angle of the polished substrate was evaluated to be 92.4°. Acid corrosion significantly changed the surface morphology of a substrate. Many graphite layers were exposed on the surface under the effect of ultrasonic and acid corrosion treatment. Moreover, the water contact angle of the acid-corroded substrate reduced to 70.3°. Clearly, acid corrosion improved the specific surface area and hydrophilicity of the graphite substrate. This favored the growth of MoS_2_ on graphite and the formation of a relatively stable structure. More importantly, the graphite substrates could be activated by nitric acid to increase the content of the oxygen-containing functional groups on the graphite substrate surface. The oxygen-containing functional groups could improve the hydrophilicity of graphite substrates, which is consistent with the contact angle measurements [42].

**Figure 2:**
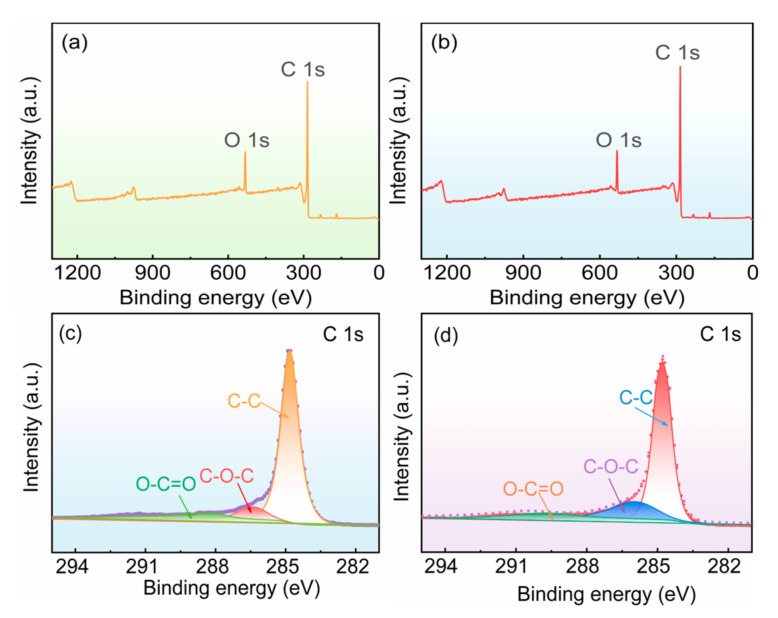
XPS spectra of polished graphite substrate and acid-corroded graphite substrate: (**a**,**b**) total spectra; (**c**,**d**) C1s spectra.

Figure 2 shows XPS spectra of polished graphite and acid-corroded graphite. As can be seen from Figure 2a,b, the polished and acid-corroded graphite substrates contained C and O elements. The C1s spectra of the polished and acid-corroded graphite substrates are shown in Figure 2c,d. The obvious peaks at 284.8 eV are both the binding states of C-C bonds. The peak at 286.2 eV corresponds to C-O-C binding, and the peak at 288.9 eV is O-C=O binding [43]. As can be seen in Table 1, after modification with HNO_3_, the proportion of the C-C peak significantly decreased, while the proportions of C-O-C and O-C=O binding states increased. This indicated that acidification increased the content of oxygen-containing functional groups. Oxygen-containing functional groups could improve the hydrophilicity of the graphite substrates, which is consistent with the results in Figure 1.

**Figure 3:**
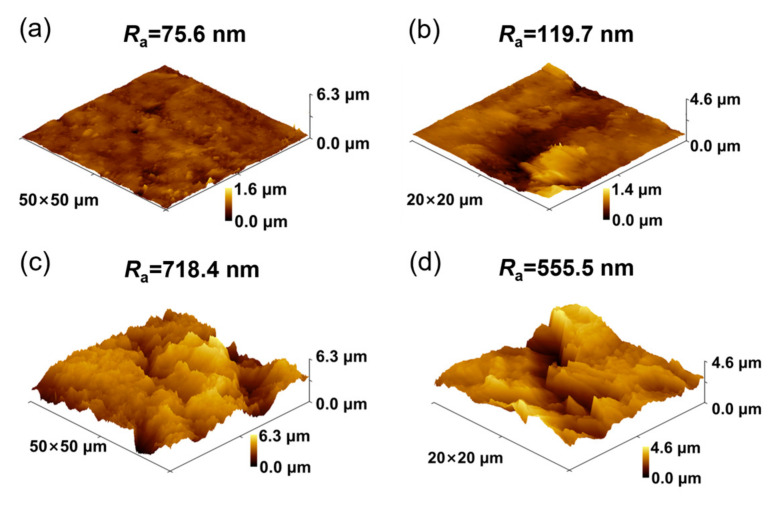
(**a**,**b**) AFM images of polished graphite substrate; (**c**,**d**) AFM images of acid-corroded graphite substrate.

Figure 3a,b show the 3D surface morphology of the polished graphite. As can be seen from the AFM results in Figure 3a, the surface of the polished graphite was relatively flat, with a roughness *R*_a_ of 75.6 nm and a surface area of 2.647 × 10^−9^ m^2^. Figure 3c,d show the 3D morphology and roughness of graphite surface after HNO_3_ acidification. The roughness *R*_a_ and surface area of graphite increased to 718.4 nm and 3.84 × 10^−9^ m^2^, respectively, as shown in Figure 3c, due to the formation of a fold layer on the surface. The fold layer increased the surface area of the graphite and provided more sites for MoS_2_ deposition on the surface.

**Figure 4:**
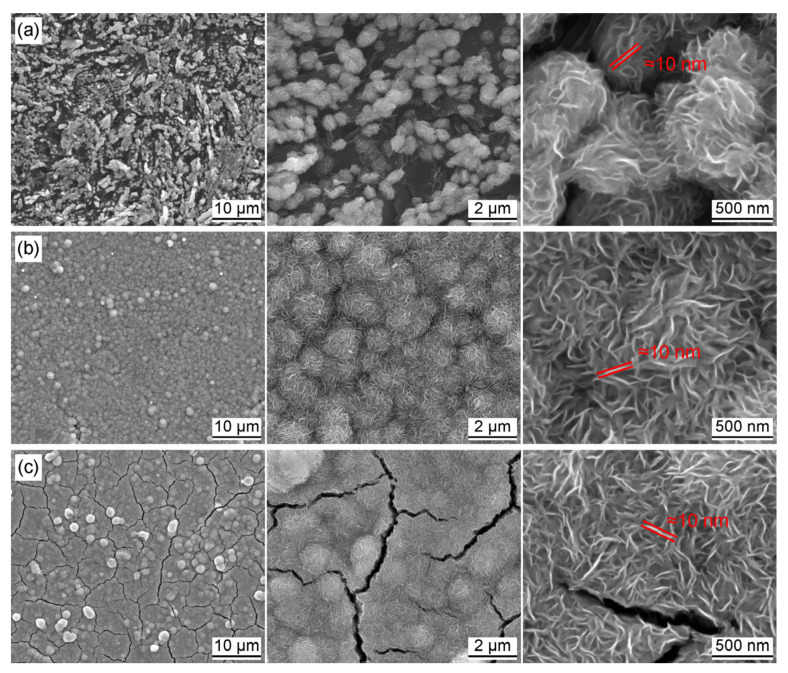
SEM images of prepared MoS_2_@Gr electrodes: (**a**) MoS_2_@Gr-0.05; (**b**) MoS_2_@Gr-0.10; (**c**) MoS_2_@Gr-0.15.

The microstructures of MoS_2_@Gr electrodes were characterized via SEM. As shown in Figure 4, nano-MoS_2_ was successfully deposited on the graphite substrate surface, while the reactant concentration had a great impact on the distribution morphology of the MoS_2_ catalyst layers. It can be seen from Figure 4a that spherical MoS_2_ particles were distributed on the substrate surface, and these flower spherical structures were not fully formed. The graphite substrate surfaces were not completely covered by MoS_2_ particles, and graphite substrates could still be observed in some locations. The main reason for this is that the low concentration of the reactant prevented the formation of enough MoS_2_ particles to cover the surface of graphite substrate. In contrast, the MoS_2_ layer was uniformly distributed on the electrode surface of MoS_2_@Gr-0.10, as shown in Figure 4b. At high magnification, it can be seen that the deposition layer was also composed of flower spherical MoS_2_ particles. Each spherical MoS_2_ particle consisted of lots of nano-MoS_2_ sheets with a thickness of about 10 nm. These nanosheets interspersed with each other and continuously accumulated and thickened to form regular microspheres. A higher reactant concentration was disadvantageous for nano-MoS_2_ catalyst layer deposition. Cracks were clearly observed on the MoS_2_@Gr-0.15 electrode surface because the higher reactant concentration les to an increase in the deposition layer thickness. A thicker deposition layer on the electrode surface can easily crack during cleaning and drying process. It can also be observed from Figure 4 that the thickness of the MoS_2_ flakes in each sample was close to 10 nm, and most of these nanoflakes vertically grew on the graphite substrate. According to Jaramillo et al. [44], the edges of folded flakes are more easily exposed, promoting the exposure of unsaturated sulfur atoms and improving hydrogen evolution activity.

**Figure 5:**
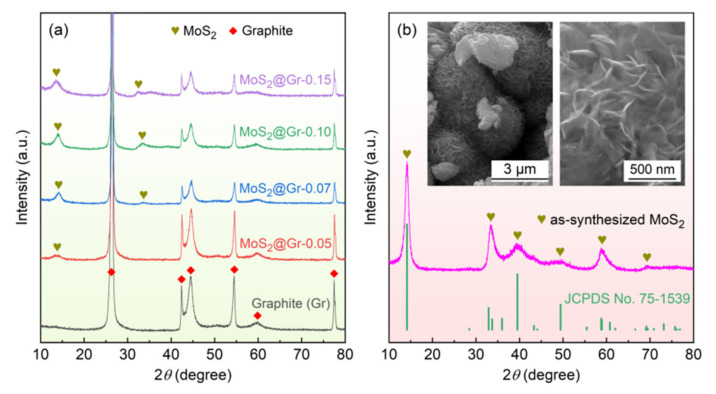
XRD patterns of prepared samples: (**a**) graphite substrate and MoS_2_@Gr electrodes; (**b**) as-synthesized pure MoS_2_ powders.

The XRD patterns obtained from the graphite substrate, MoS_2_@Gr, and pure MoS_2_ powders are shown in Figure 5. The diffraction peaks of graphite were the characteristic peaks of graphite substrates, and no other impurity peaks could be observed in the substrate, as shown in Figure 5a. In comparison, small diffraction peaks at 2*θ* ≈ 14° and 2*θ* ≈ 33° corresponding to MoS_2_ were detected in the MoS_2_@Gr samples. The diffraction peak intensity of MoS_2_ became stronger with increasing reactant concentration, indicating that MoS_2_ was successfully deposited on the graphite substrate surface. These results are consistent with the SEM results. In addition, the SEM images and XRD pattern of the pure MoS_2_ powders are compared in Figure 5b. The analysis of the results indicated that there was no trace of impurities in the as-synthesized MoS_2_ powders, and the powder sample consisted of spheroidal particles with an average diameter of about 3 μm. The high-magnification SEM image illustrated that each spheroidal particle was also formed of nano-MoS_2_ sheets. The morphological features were similar to those of the deposited MoS_2_ layers shown in Figure 4.

**Figure 6:**
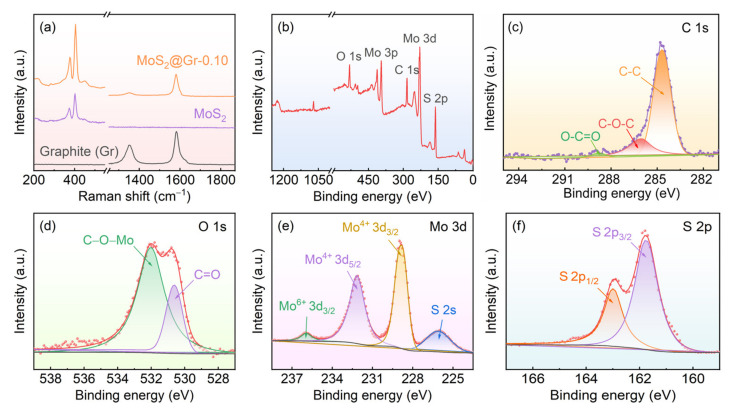
(**a**) Raman spectra of graphite substrate, MoS_2_, and MoS_2_@Gr-0.10 sample; (**b**–**f**) XPS spectra of MoS_2_@Gr-0.10.

Raman measurements of the graphite substrate after acidification, pure MoS_2_, and MoS_2_@Gr-0.10 were carried out to further characterize the structural features of the samples, and the results are shown in Figure 6a. Two sharp peaks at 1347 cm^−1^ and 1596 cm^−1^ of the graphite substrate after the acidification sample represent D and G peaks, respectively, which reflect the degree of graphite disorder and order, respectively [45]. The D peak indicates a defective peak caused by the vibrations of sp^3^-hybridized carbon atoms in the amorphous carbon in a sample, and the G peak represents a graphite peak generated by vibrating sp^2^-hybridized carbon atoms in graphitic carbon [46]. The peaks at 378 cm^−1^ and 403 cm^−1^ for the pure MoS_2_ sample indicated the existence of 2H phases of MoS_2_ [47,48]. The two peaks correspond to two different vibrational modes of in-plane and out-of-plane Mo-S, where the frequency differences between the vibrational modes mean that MoS_2_ nanosheets have a multilayer structure [49]. Both graphite and MoS_2_ peaks could be observed in the MoS_2_@Gr-0.10 sample, also indicating that the MoS_2_ catalyst layer was successfully deposited on the graphite substrate.

XPS testing was used to characterize the surface elemental composition and valence bonding state of the MoS_2_@Gr-0.10 sample, and the results are shown in Figure 6b–f. As can be seen in Figure 6b that the MoS_2_@Gr-0.10 sample was mainly composed of C, O, Mo, and S elements. The carbon mainly originated from the graphite substrate. In Figure 6d, two overlapped peaks can be observed. The peak at 530.7 eV indicates C=O bonding, and the peak at 532.1 eV indicates C-O-Mo bonding. According to Lu et al. [50], the presence of C-O-Mo bonding indicates that the chemical interaction between MoS_2_ and graphite substrate occurs through C-O-Mo. This makes the MoS_2_ and graphite substrate tightly bonded to each other, which facilitates the structural stability of catalyst layers. The main peaks at 229.6 eV and 232.8 eV shown in Figure 6e belong to Mo 3d_5/2_ and Mo 3d_3/2_ orbitals, respectively, corresponding to Mo^4+^ [51,52]. Moreover, there was a weak peak corresponding to Mo^6+^ at 235.8 eV, indicating that MoS_2_ caused mild oxidation in the air. The peaks at 161.2 eV and 163.6 eV in Figure 6f belong to S 2p_3/2_ and S 2p_1/2_, respectively, which correspond to the −2 valence in the sulfur of MoS_2_, reflecting the formation of the 2H phase of MoS_2_ [53,54].

### 3.2. Electrochemical HER Performance

**Figure 7:**
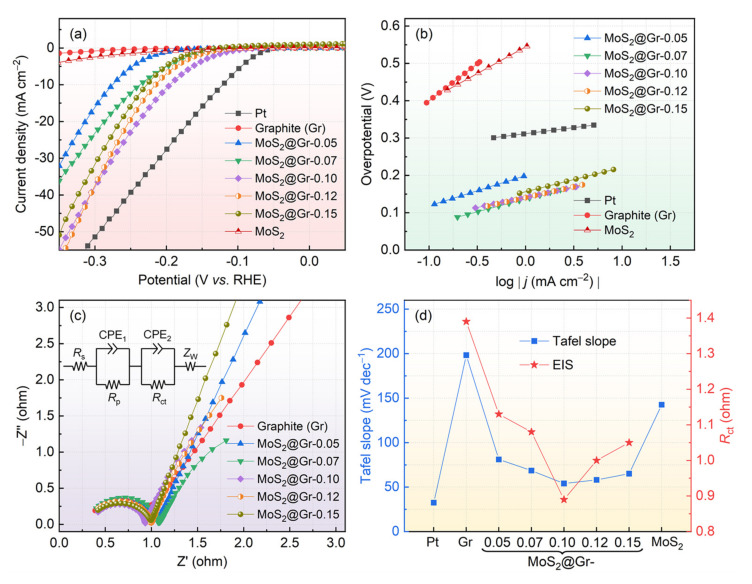
(**a**) Polarization curves of Pt and MoS_2_@Gr samples; (**b**) Tafel slops of Pt and MoS_2_@Gr samples; (**c**) Nyquist curve of MoS_2_@Gr samples; (**d**) comparison of Tafel slopes and *R*_ct_.

The electrochemical HER property of the prepared electrodes was tested in a typical three-electrode system. For comparison, the electrocatalytic properties of Pt and pure MoS_2_@Gr samples were also measured under the same conditions. The LSV curves of the samples are shown in Figure 7a. As shown, these samples produced different electrocatalytic performances. The graphite substrate had almost no catalytic activity in the HER process, and the pure MoS_2_ also exhibited poor catalytic activity. In contrast, the prepared MoS_2_@Gr electrodes showed good HER catalytic performance. The overpotential of the MoS_2_@Gr electrodes at a current density of 10 mA·cm^−2^ first increased and then decreased with the increase in the reactant concentration, and the MoS_2_@Gr-0.10 sample had the lowest initial HER overvoltage of 113 mV. The variation in the HER catalytic properties of the MoS_2_@Gr electrodes was mainly affected by the microstructural difference caused by the reactant concentration (see Figure 4). For the MoS_2_@Gr-0.05 and MoS_2_@Gr-0.07 samples, the exposed MoS_2_ edges were not enough on the electrode surface, resulting in poor HER catalytic performance. With respect to the MoS_2_@Gr-0.12 and MoS_2_@Gr-0.15 samples, excessive MoS_2_ nanosheets accumulated on the electrode surface, leading to the edge coverage of active sites and lower conductivity of catalyst layer. The Pt electrode had the best general performance. In this study, nano-MoS_2_ was successfully deposited on a graphite substrate, and its catalytic activity for HER may be further improved via structural modification [55], heteroatoms doping [56], etc.

Under ideal conditions, Tafel slopes usually represent the intrinsic properties of electrocatalysts and reaction kinetic issues that can well explain the active sites of catalysts and enable analysis of HER mechanisms [57]. According to Lin et al. [58,59], a smaller Tafel slope represents a faster current density growth when the overpotential increases by an equal amount. As shown in Figure 7b, the Tafel slopes of the MoS_2_@Gr-0.05, MoS_2_@Gr-0.07, MoS_2_@Gr-0.10, MoS_2_@Gr-0.12, and MoS_2_@Gr-0.15 electrodes were calculated as 81.1, 68.6, 54.1, 58.1, and 65.1 mV·dec^−1^, respectively. Clearly, the MoS_2_@Gr-0.10 electrode had the lowest Tafel slope value after that of the Pt electrode.

The electron exchange process in electrochemical reactions usually occurs at an electrode surface between the electrons in an electrode and the ions in the solution, and this process often occurs in redox reactions. Therefore, EIS tests were conducted to describe the kinetic process of electron transfer rate at the electrode surface. A lower charge transfer resistance means faster HER kinetic properties. As shown in Figure 7c, Nyquist plots are presented as semicircles in the high-frequency region and slanted lines in the low-frequency region. These electrochemical measurements are represented by a fitted circuit in Figure 7c, where *R*_s_, *R*_p_, and *R*_ct_ represent the resistance, electrode porosity, and charge transfer resistance of the electrolyte solution, respectively; CPE is a constant phase angle element that represents a solid double-layer capacitance of an electrode in real situations; and Z_w_ represents ion diffusion process in liquid solution.

To better understand the electrochemical catalytic activity of each electrode, Tafel slopes and *R*_ct_ values are compared in Figure 7d. It can be observed that Pt has the lowest Tafel slope value. For the other samples, the Tafel slope values present a trend of first decreasing and then increasing. Additionally, the *R*_ct_ values first decrease and then increase, which is consistent with the Tafel slope values. The much smaller *R*_ct_ values of the MoS_2_@Gr samples compared with those of the graphite substrate suggested that the MoS_2_@Gr samples had superior ion diffusion behavior. Among these MoS_2_@Gr electrodes, the MoS_2_@Gr-0.10 sample exhibited the best HER catalytic performance. Clearly, the microstructure of deposition catalyst layer was the decisive factor for HER catalytic activity. The microstructure of catalyst layer could be conveniently controlled by changing the hydrothermal reaction conditions, such as reactant concentration, reaction temperature, reaction time, and so on.

**Figure 8:**
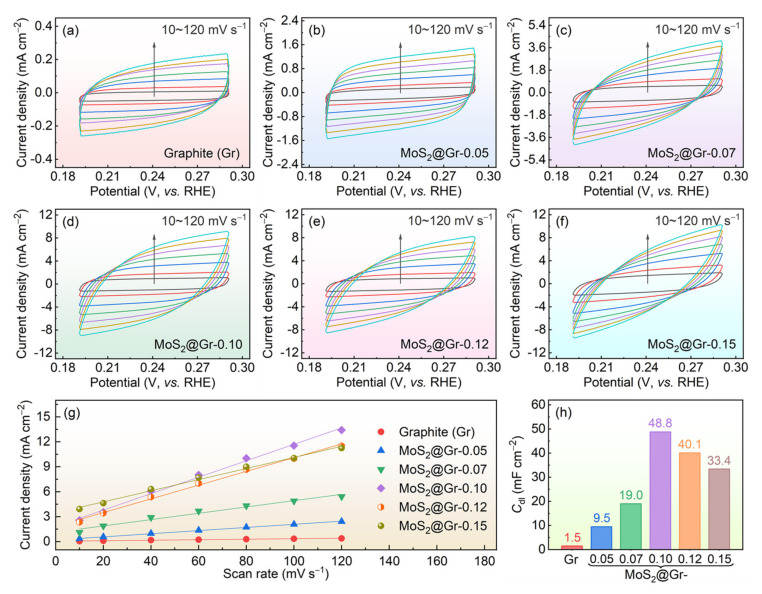
(**a**–**f**) CV curves of the samples at different scan rates; (**g**) linear fitting curve of current density and scanning rate; (**h**) comparison of *C*_dl_ values.

Generally, there is a linear relationship between *C*_dl_ and electrochemically active surface area (ECSA) [60]. Electrochemical CV curves were obtained in the voltage range of 0.15–0.35 V (vs. RHE) at multiple scan rates (10–120 mV·s^−1^), and the results are shown in Figure 8a–f. According to Feng et al. [61], *C*_dl_ can be estimated by plotting the differences in current density (∆*j* = *j*_a_ − *j*_c_) at 0.241 V as a function of the scan rate. Based on the slopes of the line shown in Figure 8g, the *C*_dl_ values were calculated and are displayed in Figure 8h. By comparing the *C*_dl_ values of MoS_2_@Gr samples, we concluded that the ECSA of MoS_2_@Gr-0.10 was larger than that of the other samples. The higher *C*_dl_ value of the MoS_2_@Gr-0.10 electrode was closely related to its microstructure feature: the increase in MoS_2_ edges on the MoS_2_@Gr-0.10 electrode remarkably promoted its HER process.

### 3.3. Cycling Stability

**Figure 9:**
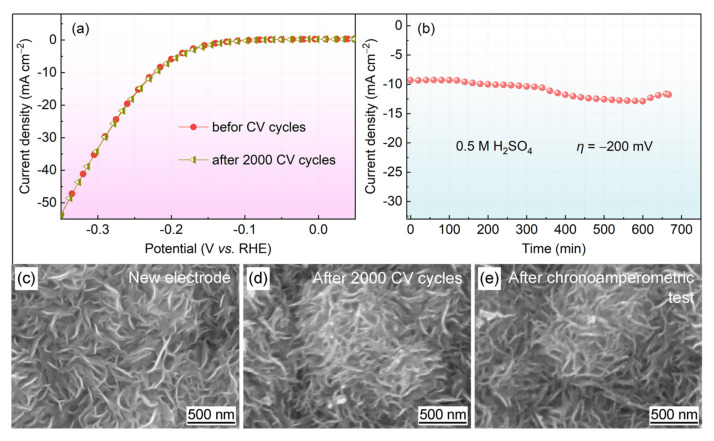
(**a**) Polarization curves of the MoS_2_@Gr-0.10 electrode before and after 2000 CV cycles; (**b**) chronoamperometric curve of the MoS_2_@Gr-0.10 electrode; (**c**–**e**) SEM images of the MoS_2_@Gr-0.10 electrode.

In addition to electrocatalytic performance, stability is an important criterion to evaluate the performance of electrocatalysts. The electrochemical stability of the MoS_2_@Gr-0.10 electrode was determined via long-term CV tests. As shown in Figure 9a, after 2000 CV cycles, the change in the polarization curve of the sample was almost negligible. There was no loss of any current density, and the curves before and after CV cycles overlapped relatively well. This indicates that the MoS_2_@Gr-0.10 catalyst had very good stability and excellent HER performance during a long electrochemical time in 0.5 M H_2_SO_4_ solution. After that, the electrode was subjected to a chronoamperometry, and the results are shown in Figure 9b. After 660 min durability experiments, the current density of the acidic solution showed variances but with small fluctuations.

The good cycling performance of the MoS_2_@Gr-0.10 electrode was mainly due to its structural stability during electrochemical cycling process. As illustrated in Figure 9d, nano-MoS_2_ was still clearly visible on the electrode surface after 2000 CV cycling tests. There was no obvious exfoliation of MoS_2_ sheets, indicating that the catalyst layer had a good adhesion force with the graphite substrate. This mainly benefitted from the inherent stability of graphite and the in situ growth of MoS_2_ on the graphite substrate. In summary, based on the microstructural characterization of the materials and the comparison of the results of HER performance, we found that the MoS_2_@Gr-0.10 electrocatalyst had a good morphological structure and excellent HER performance. Although the in situ growth of MoS_2_ on graphite substrates is a simple and rapid process, its activity can be further improved as a catalyst for hydrogen evolution. In subsequent work, the surface area of graphite can be further increased, the size of MoS_2_ can be decreased, and metal atoms can be doped to improve the hydrogen evolution activity of the catalysts. The HER performance of MoS_2_@Gr was compared with that of the reported electrocatalysts, and the results are tabulated in Table 2.

## 4. Conclusions

(1) Nano-MoS_2_ was successfully deposited on the surface of a graphite substrate via a one-step hydrothermal method, and the microstructure of the MoS_2_ layers could be controlled by changing concentration of reactant.
(2) A dense and uniform MoS_2_ layer was the key factor to improve the HER catalytic activity of the MoS_2_@Gr electrodes. However, a higher reactant concentration led to an increase in the deposited MoS_2_ layer thickness, which resulted in edge coverage of active sites and a decrease in the conductivity of the catalyst.
(3) The MoS_2_@Gr-0.10 electrode showed the best electrochemical performance with an overpotential of 196 mV at 10 mA·cm^−2^ and a Tafel slope of 54.1 mV·dec^−1^.
(4) There was no catalytic activity loss of the MoS_2_@Gr-0.10 electrode after 2000 CV cycles, and the electrode exhibited good stability performance.

## Figures and Tables

**Table 1:** Area percentages of peaks in the C1s spectra of polished and acid-corroded graphite substrates.

Sample	Area Percentage (%)		
	C-C	C-O-C	O-C=O
Polished graphite substrate	82.24	9.33	8.43
Acid-corroded graphite substrate	69.04	21.58	9.38

**Table 2:** Comparison of differences between MoS_2_@Gr electrode and previous MoS_2_ electrode preparation methods.

Catalyst	Synthesis Approach	Nafion	*η*_10_ (mV vs. RHE)	Tafel (mV·dec^−1^)	Ref.
MoS_2_@Fe/Ni-MOF_600_	Hydrothermal	Yes	214	170.7	[62]
GC/MoS_2_ film	Electrodeposition	No	202	48	[63]
MoS_2−x_	Solvothermal	Yes	191	67	[64]
MoS_2_-MoO_2_	CVD	No	198	66.8	[65]
MoS_2_-MoO_2_	Hot-injection method	Yes	210	129	[66]
MoS_2_/SSF	Hydrothermal	No	151	55.7	[67]
MoS_2_/G	Sulfurization treatment	Yes	208	59	[68]
MoS_2_/Gr	Hydrothermal	No	196	54.1	This work

## Data Availability

Data sharing is not applicable to this article as no new data were created or analyzed in this study.

## References

[1] Wang J., Kong H., Zhang J., Hao Y., Shao Z., Ciucci F. (2021). Carbon-based electrocatalysts for sustainable energy applications. Carbon-based electrocatalysts for sustainable energy applications. Prog. Mater. Sci..

[2] Abbasi K.R., Shahbaz M., Zhang J., Irfan M., Alvarado R. (2022). Analyze the environmental sustainability factors of China: The role of fossil fuel energy and renewable energy. Renew. Energy.

[3] Moustakas K., Loizidou M., Rehan M., Nizami A.S. (2020). A review of recent developments in renewable and sustainable energy systems: Key challenges and future perspective. Renew. Sustain. Energy Rev..

[4] Gapp E., Pfeifer P. (2023). Membrane reactors for hydrogen production from renewable energy sources. Curr. Opin. Green Sustain. Chem..

[5] Abdin Z., Zafaranloo A., Rafiee A., Mérida W., Lipiński W., Khalilpour K.R. (2020). Hydrogen as an energy vector. Renew. Sustain. Energy Rev..

[6] Bhutto A.W., Bazmi A.A., Zahedi G. (2013). Underground coal gasification: From fundamentals to applications. Prog. Energy Combust..

[7] Yi L., Fan Y., Yang R., Zhu R., Zhu Z., Hu J. (2022). Fabrication and optimization of CdS photocatalyst using nature leaf as biological template for enhanced visible-light photocatalytic hydrogen evolution. Catal. Today.

[8] Daulbayev C., Sultanov F., Korobeinyk A.V., Yeleuov M., Azat S., Bakbolat B., Umirzakov A., Mansurov Z. (2021). Bio-waste-derived few-layered graphene/SrTiO_3_/PAN as efficient photocatalytic system for water splitting. Appl. Surf. Sci..

[9] Daulbayev C., Sultanov F., Bakbolat B., Daulbayev O. (2020). 0D, 1D and 2D nanomaterials for visible photoelectrochemical water splitting. A Review. Int. J. Hydrogen Energy.

[10] Sultanov F., Daulbayev C., Azat S., Kuterbekov K., Bekmyrza K., Bakbolat B., Bigaj M., Mansurov Z. (2020). Influence of Metal Oxide Particles on Bandgap of 1D Photocatalysts Based on SrTiO_3_/PAN Fibers. Nanomaterials.

[11] Wang M., Wang Z., Gong X., Guo Z. (2014). The intensification technologies to water electrolysis for hydrogen production—A review. Renew. Sustain. Energy Rev..

[12] Thomas J.M., Edwards P.P., Dobson P.J., Owen G.P. (2020). Decarbonizing energy: The developing international activity in hydrogen technologies and fuel cells. J. Energy Chem..

[13] Hosseini S.E., Wahid M.A. (2016). Hydrogen production from renewable and sustainable energy resources: Promising green energy carrier for clean development. Renew. Sustain. Energy Rev..

[14] Hu J., Zhu S., Liang Y., Wu S., Li Z., Luo S., Cui Z. (2021). Self-supported Ni_3_Se_2_@NiFe layered double hydroxide bifunctional electrocatalyst for overall water splitting. J. Colloid Interface Sci..

[15] Li S., Sun J., Guan J. (2021). Strategies to improve electrocatalytic and photocatalytic performance of two-dimensional materials for hydrogen evolution reaction. Chin. J. Catal..

[16] Jiang G., Zhang C., Liu X., Bai J., Xu M., Xu Q., Li Y., Long L., Zhang G., Li S. (2022). Electrocatalytic hydrogen evolution of highly dispersed Pt/NC nanoparticles derived from porphyrin MOFs under acidic and alkaline medium. Int. J. Hydrogen Energy.

[17] Li J., Liu H.X., Gou W., Zhang M., Xia Z., Zhang S., Chang C.R., Ma Y., Qu Y. (2019). Ethylene-glycol ligand environment facilitates highly efficient hydrogen evolution of Pt/CoP through proton concentration and hydrogen spillover. Energy Environ. Sci..

[18] Anantharaj S., Karthik P.E., Subramanian B., Kundu S. (2016). Pt nanoparticle anchored molecular self-assemblies of DNA: An extremely stable and efficient HER electrocatalyst with ultralow Pt content. ACS Catal..

[19] Laszczyńska A., Tylus W., Szczygieł I. (2021). Electrocatalytic properties for the hydrogen evolution of the electrodeposited Ni–Mo/WC composites. Int. J. Hydrogen Energy.

[20] Yang Y., Yao H., Yu Z., Islam S.M., He H., Yuan M., Yue Y., Xu K., Hao W., Sun G. (2019). Hierarchical nanoassembly of MoS_2_/Co_9_S_8_/Ni_3_S_2_/Ni as a highly efficient electrocatalyst for overall water splitting in a wide pH range. J. Am. Chem. Soc..

[21] Yin W., He D., Bai X., Yu W.W. (2019). Synthesis of tungsten disulfide quantum dots for high-performance supercapacitor electrodes. J. Alloys Compd..

[22] Pataniya P.M., Sumesh C.K. (2021). Enhanced electrocatalytic hydrogen evolution reaction by injection of photogenerated electrons in Ag/WS_2_ nanohybrids. Appl. Surf. Sci..

[23] Shajaripour Jaberi S.Y., Ghaffarinejad A., Khajehsaeidi Z., Sadeghi A. (2023). The synthesis, properties, and potential applications of CoS_2_ as a transition metal dichalcogenide (TMD). Int. J. Hydrogen Energy.

[24] Zhang H.C., Li Y.J., Zhang G.X., Wan P.B., Xu T.H., Wu X.C., Sun X.M. (2014). Highly crystallized cubic cattierite CoS_2_ for electrochemically hydrogen evolution over wide pH range from 0 to 14. Electrochim. Acta.

[25] Guo Y.J., Guo D., Ye F., Wang K., Shi Z.Q. (2017). Synthesis of lawn-like NiS_2_ nanowires on carbon fiber paper as bifunctional electrode for water splitting. Int. J. Hydrogen Energy.

[26] Liu P., Li J., Lu Y., Xiang B. (2018). Facile synthesis of NiS_2_ nanowires and its efficient electrocatalytic performance for hydrogen evolution reaction. Int. J. Hydrogen Energy.

[27] Ma Q.Y., Hu C.Y., Liu K.L., Hung S.F., Ou D.H., Chen H.M., Fu G., Zheng N.F. (2017). Identifying the electrocatalytic sites of nickel disulfide in alkaline hydrogen evolution reaction. Nano Energy.

[28] Li W., Bi R., Liu G.X., Tian Y., Zhang L. (2018). 3D Interconnected MoS_2_ with enlarged interlayer spacing grown on carbon nanofibers as a flexible anode toward superior sodium-ion batteries. ACS Appl. Mater. Interfaces.

[29] Zhao L.P., Qi L., Wang H. (2015). MoS_2_–C/graphite, an electric energy storage device using Na^+^-based organic electrolytes. RSC Adv..

[30] Veerasubramani G.K., Park M.S., Nagaraju G., Kim D.W. (2019). Unraveling the Na-ion storage performance of a vertically aligned interlayer-expanded two-dimensional MoS_2_@C@MoS_2_ heterostructure. J. Mater. Chem. A.

[31] Li X., Zhu H.W. (2015). Two-dimensional MoS_2_: Properties, preparation, and applications. J. Mater..

[32] Liu Q.L., Shi H.D., Yang T.Y., Yang Y., Wu Z.S., Yu J.Q., Silva S.R.P., Liu J. (2019). Sequential growth of hierarchical N-doped carbon-MoS_2_ nanocomposites with variable nanostructures. J. Mater. Chem. A.

[33] Gong F.L., Ye S., Liu M.M., Zhang J.W., Gong L.H., Zeng G., Meng E., Su P., Xie K.F., Zhang Y.H. (2020). Boosting electrochemical oxygen evolution over yolk-shell structured O–MoS_2_ nanoreactors with sulfur vacancy and decorated Pt nanoparticles. Nano Energy.

[34] Wu Z., Fang B., Wang Z., Wang C., Wilkinson D.P. (2013). MoS_2_ Nanosheets: A designed structure with high active site density for the hydrogen evolution reaction. Acs Catal..

[35] Lin H., Zhang K., Yang G., Li Y., Liu X., Chang K., Xuan Y., Ye J. (2020). Ultrafine nano 1T-MoS_2_ monolayers with NiO_x_ as dual co-catalysts over TiO_2_ photoharvester for efficient photocatalytic hydrogen evolution. Appl. Catal. B Environ..

[36] Fu W., Zhao Y., Wang H., Chen X., Liu K., Zhang K., Wei Q., Wang B. (2022). Study on preparation, photocatalytic performance and degradation mechanism of polymeric carbon nitride/Pt/nano-spherical MoS_2_ composite. J. Phys. Chem. Solids.

[37] Yu Y.F., Huang S.Y., Li Y.P., Steinmann S.N., Yang W., Cao L.Y. (2014). Layer-dependent electrocatalysis of MoS_2_ for hydrogen evolution. Nano Lett..

[38] Zhang X., Jia F., Song S. (2021). Recent advances in structural engineering of molybdenum disulfide for electrocatalytic hydrogen evolution reaction. Chem. Eng. J..

[39] Nguyen D.C., Luyen Doan T.L., Prabhakaran S., Tran D.T., Kim D.H., Lee J.H., Kim N.H. (2021). Hierarchical Co and Nb dual-doped MoS_2_ nanosheets shelled micro-TiO_2_ hollow spheres as effective multifunctional electrocatalysts for HER, OER, and ORR. Nano Energy.

[40] Tang B.S., Yu Z.G., Seng H.L., Zhang N.D., Liu X.X., Zhang Y.W., Yang W.F., Gong H. (2018). Simultaneous edge and electronic control of MoS_2_ nanosheets through Fe doping for an efficient oxygen evolution reaction. Nanoscale.

[41] Wang M., Jian K., Lv Z., Li D., Fan G., Zhang R., Dang J. (2021). MoS_2_/Co_9_S_8_/MoC heterostructure connected by carbon nanotubes as electrocatalyst for efficient hydrogen evolution reaction. J. Mater. Sci. Technol..

[42] Wang Y., Zhou W., Gao J.H., Ding Y.N., Kou K.K. (2019). Oxidative modification of graphite felts for efficient H_2_O_2_ electrogeneration: Enhancement mechanism and long-term stability. J. Electroanal. Chem..

[43] Zhou Y., Liu G., Zhu X., Guo Y. (2017). Ultrasensitive NO_2_ gas sensing based on rGO/MoS_2_ nanocomposite film at low temperature. Sens. Actuat. B Chem..

[44] Jaramillo T.F., Jørgensen K.P., Bonde J., Nielsen J.H., Horch S., Chorkendorff I. (2007). Identification of active edge sites for electrochemical H_2_ evolution from MoS_2_ nanocatalysts. Science.

[45] Rowley-Neale S.J., Brownson D.A.C., Smith G.C., Sawtell D.A.G., Kelly P.J., Banks C.E. (2015). 2D nanosheet molybdenum disulphide (MoS_2_) modified electrodes explored towards the hydrogen evolution reaction. Nanoscale.

[46] Ferrari A.C., Robertson J. (2000). Interpretation of Raman spectra of disordered and amorphous carbon. Phys. Rev. B.

[47] Patel P.P., Velikokhatnyi O.I., Ghadge S.D., Hanumantha P.J., Datta M.K., Kuruba R., Gattu B., Shanthi P.M., Kumta P.N. (2018). Electrochemically active and robust cobalt doped copper phosphosulfide electro-catalysts for hydrogen evolution reaction in electrolytic and photoelectrochemical water splitting. Int. J. Hydrogen Energy.

[48] Vikraman D., Hussain S., Ali M., Karuppasamy K., Santhoshkumar P., Hwang J.H., Jung J., Kim H.S. (2021). Theoretical evaluation and experimental investigation of layered 2H/1T-phase MoS_2_ and its reduced graphene-oxide hybrids for hydrogen evolution reactions. J. Alloys Compd..

[49] Shilpa R., Sibi K.S., Pai R.K., Sarath Kumar S.R., Rakhi R.B. (2022). Electrocatalytic water splitting for efficient hydrogen evolution using molybdenum disulfide nanomaterials. Mater. Sci. Eng. B.

[50] Lu H., Tian K., Bu L.M., Huang X., Li X.Y., Zhao Y., Wang F., Bai J., Gao L., Zhao J.Q. (2021). Synergistic effect from coaxially integrated CNTs@MoS_2_/MoO_2_ composite enables fast and stable lithium storage. J. Energy Chem..

[51] Guan X.B., Zhao L.P., Zhang P., Liu J., Song X.F., Gao L. (2020). Electrode material of core-shell hybrid MoS_2_@C/CNTs with carbon intercalated few-layer MoS_2_ nanosheets. Mater. Today Energy.

[52] Jiang Y., Guo Y., Lu W., Feng Z., Xi B., Kai S., Zhang J., Feng J., Xiong S. (2017). Rationally incorporated MoS_2_/SnS_2_ nanoparticles on graphene sheets for lithium-ion and sodium-ion batteries. ACS Appl. Mater. Interfaces.

[53] Hong Z.A., Hong W.T., Wang B.C., Cai Q., He X., Liu W. (2023). STable 1T –2H MoS_2_ heterostructures for efficient electrocatalytic hydrogen evolution. Chem. Eng. J..

[54] Wang D.Z., Su B.Y., Jiang Y., Li L., Ng B.K., Wu Z.Z., Liu F. (2017). Polytype 1T/2H MoS_2_ heterostructures for efficient photoelectrocatalytic hydrogen evolution. Chem. Eng. J..

[55] Xu Y., Qu J.T., Li Y., Zhu M.Y., Liu Y., Zheng R., Cairney J.M., Li W.X. (2020). Bridging metal-ion induced vertical growth of MoS_2_ and overall fast electron transfer in (C, P)_3_N_4_-M (Ni^2+^, Co^2+^)-MoS_2_ electrocatalyst for efficient hydrogen evolution reaction. Sustain. Mater. Techno..

[56] Wu L.Q., Xu X.B., Zhao Y.Q., Zhang K.Y., Sun Y., Wang T.T., Wang Y.Q., Zhong W., Du Y. (2017). Mn doped MoS_2_/reduced graphene oxide hybrid for enhanced hydrogen evolution. Appl. Surf. Sci..

[57] Anantharaj S., Noda S. (2022). How properly are we interpreting the Tafel lines in energy conversion electrocatalysis?. Mater. Today Energy.

[58] Lin Y., Pan Y., Zhang J., Chen Y.J., Sun K., Liu Y., Liu C. (2016). Graphene oxide co-doped with nitrogen and sulfur and decorated with cobalt phosphide nanorods: An efficient hybrid catalyst for electrochemical hydrogen evolution. Electrochim. Acta.

[59] Ye J.B., Yu Z.T., Chen W.X., Chen Q.N., Xu S.R., Liu R. (2016). Facile synthesis of molybdenum disulfide/nitrogen-doped graphene composites for enhanced electrocatalytic hydrogen evolution and electrochemical lithium storage. Carbon.

[60] Anantharaj S., Ede S.R., Karthick K., Sam Sankar S., Sangeetha K., Karthik P.E., Kundu S. (2018). Precision and correctness in the evaluation of electrocatalytic water splitting: Revisiting activity parameters with a critical assessment. Energy Environ. Sci..

[61] Feng J.H., Zhou H., Wang J.P., Bian T., Shao J.X., Yuan A.H. (2018). MoS_2_ supported on MOF-derived carbon with core-shell structure as efficient electrocatalysts for hydrogen evolution reaction. Int. J. Hydrogen Energy.

[62] Lin Z., Feng T., Ma X., Liu G. (2023). Fe/Ni bi-metallic organic framework supported 1T/2H MoS_2_ heterostructures as efficient bifunctional electrocatalysts for hydrogen and oxygen evolution. Fuel.

[63] Ambrosi A., Pumera M. (2016). Templated electrochemical fabrication of hollow molybdenum sulfide microstructures and nanostructures with catalytic properties for hydrogen production. ACS Catal..

[64] Zhao M., Ma X., Yan S., Xiao H., Li Y., Hu T., Zheng Z., Jia J., Wu H. (2020). Solvothermal synthesis of oxygen-incorporated MoS_2-x_ nanosheets with abundant undercoordinated Mo for efficient hydrogen evolution. Int. J. Hydrogen Energy.

[65] Kang H., Youn J.S., Oh I., Manavalan K., Jeon K.J. (2020). Controllable atomic-ratio of CVD-grown MoS_2_-MoO_2_ hybrid catalyst by soft annealing for enhancing hydrogen evolution reaction. Int. J. Hydrogen Energy.

[66] Wu C.L., Huang P.C., Brahma S., Huang J.L., Wang S.C. (2017). MoS_2_-MoO_2_ composite electrocatalysts by hot-injection method for hydrogen evolution reaction. Ceram. Int..

[67] Wang H.B., Zhu H., Sun Y.S., Ma F., Chen Y.Z., Zeng D.J., Zhou L., Ma D.Y. (2021). Ultra-thin pine tree-like MoS_2_ nanosheets with maximally exposed active edges terminated at side surfaces on stainless steel fiber felt for hydrogen evolution reaction. J. Alloys Compd..

[68] Cao K., Sun S., Song A., Ba J., Lin H., Yu X., Xu C., Jin B., Huang J., Fan D. (2022). Increased 1T-MoS_2_ in MoS_2_@CoS_2_/G composite for high-efficiency hydrogen evolution reaction. J. Alloys Compd..

